# Case Report: COVID-19 Associated Renal Infarction and Ascending Aortic Thrombosis

**DOI:** 10.4269/ajtmh.20-0869

**Published:** 2020-09-10

**Authors:** Aveek Mukherjee, Raisa Ghosh, Marlene Marte Furment

**Affiliations:** Division of Internal Medicine, Rutgers Robert Wood Johnson Medical School/Saint Peter’s University Hospital, New Brunswick, New Jersey

## Abstract

Following its discovery in Wuhan, China, in December 2019, COVID-19 has attained pandemic status in mere months. It is caused by SARS-CoV-2, an enveloped beta coronavirus. This infection causes a prothrombogenic state by interplay of inflammatory mediators, and endothelial, microvascular, and possible hepatic damage and tissue tropism of the virus. This leads to frequent pulmonary and cerebral thromboembolism as well as occasional involvement of other organs. We present a 71-year-old man who initially presented with 2 weeks of fever, cough, and shortness of breath and was diagnosed with COVID-19 pneumonia. He required readmission due to worsened hypoxia and was later found to have left renal artery thrombosis with left kidney infarction, associated with an ascending aortic thrombus. He was anticoagulated and recovered uneventfully. We suggest that physicians have a high degree of suspicion to diagnose and manage the novel manifestations of this disease.

## INTRODUCTION

After its discovery in Wuhan, China, in December 2019, COVID-19 has become a pandemic in just months. COVID-19 commonly presents with fever (88.7%), cough (67.8%), fatigue (38.1%), sputum production (33.7%), shortness of breath (18.7%), and myalgia (14.9%), whereas nausea or vomiting (5%) and diarrhea (3.8%) are uncommon.^1^ COVID-19 infection has been found to be a prothrombogenic state.^2–5^ We present a 71-year-old man who initially presented with 2 weeks of fever, cough, and shortness of breath. He was diagnosed with COVID-19 multifocal pneumonia and required readmission due to worsening hypoxia. During his second hospitalization, he had left flank pain and was found to have left renal artery thrombi with left kidney infarction associated with an ascending aortic thrombus. He was anticoagulated with intravenous heparin infusion and transitioned to oral apixaban on discharge after an uneventful recovery.

## CASE

**Figure 1.**
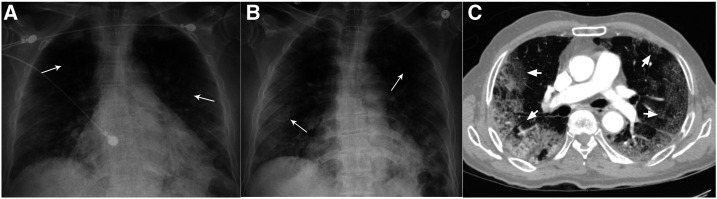
Chest radiography. (**A**) Chest X-ray on initial presentation showing bilateral peripheral patchy infiltrates (arrows). (**B**) Chest X-ray on readmission showing worsened bilateral peripheral patchy infiltrates (arrows). (**C**) Computed tomogram of the thorax showing peripheral-based extensive multi-lobar ground-glass opacities (arrows).

A 71-year-old man with no significant past medical history presented with 2 weeks of fever, cough, shortness of breath, and chest discomfort with coughing in early April 2020 at a tertiary care hospital in New Brunswick, NJ. He was exposed to his daughter who was diagnosed with COVID-19 pneumonia a week before he fell ill. On presentation, he was afebrile at 99.7°F (37.6°C), blood pressure was 134/67 mmHg, tachycardic with a pulse of 102/minute, tachypneic at 34 breaths/minute, and hypoxic at 78% breathing room air. Examination was notable for faint crackles over all lung fields. Supplemental oxygen via nasal cannula (NC) was initiated at 4 L/minute with improvement of saturation to 94%. Initial laboratory investigations revealed absolute lymphopenia and elevated D-dimer, lactate dehydrogenase (LDH), ferritin, and C-reactive protein (CRP) (Table 1). His chest X-ray showed peripheral bilateral patchy opacities (Figure 1A). A nasopharyngeal swab specimen tested positive for SARS-CoV-2 by reverse transcription–PCR. We started him on hydroxychloroquine therapy. By the third day, he was comfortable, only requiring supplemental oxygen on mobilization, and was discharged to a COVID-19 Federal Medical Station.

**Table 1:** Laboratory parameters with trends by day since initial hospital presentation

Parameter	Reference value or range	Day 1	Day 3	Day 5	Day 6	Day 8	Day 10	Day 11	Day 12	Day 13	Day 14
Absolute lymphocyte count (×10^9^/L)	1–3.5	0.26	0.73	0.60	0.87	1.54	3.21	ND	ND	ND	1.30
Platelets (×10^9^/L)	150–400	215	337	356	381	347	405	377	ND	ND	291
D-dimer (ng/mL)	0–211	317	ND	1,113	ND	439	ND	ND	576	ND	417
Lactate dehydrogenase (U/L)	140–271	424	ND	525	ND	348	ND	ND	1,105	ND	586
C-reactive protein (mg/L)	0–5	111	ND	155	ND	23	ND	ND	6	ND	39
Ferritin (ng/mL)	18–464	636	ND	856	ND	ND	ND	ND	3,640	ND	1,512
Blood urea nitrogen (mg/dL)	6–20	15	17	17	21	18	22	22	22	19	ND
Creatinine (mg/dL)	0.66–1.1	0.72	0.59	0.69	0.56	0.58	0.66	0.66	0.60	0.66	ND
Estimated glomerular filtration rate (mL/minute/1.73 m^2^)	> 60	107	> 120	112	> 120	> 120	118	118	> 120	118	ND
Sodium (mmol/L)	136–145	133	133	134	137	134	134	137	138	133	ND
Potassium (mmol/L)	3.5–5.1	3.9	4.1	4.3	4.6	4.3	4.5	5.2	5.2	4.6	ND
Chloride (mmol/L)	99–112	97	98	96	99	100	97	97	96	98	ND
Bicarbonate (mmol/L)	21–33	26	25	27	26	27	22	27	29	28	ND
Urine output (mL/day)	400–3,000	1,100	1,400	1,200	1,400	1,200	1,400	1,300	1,200	1,500	1,200

ND = not done.

**Figure 2.**
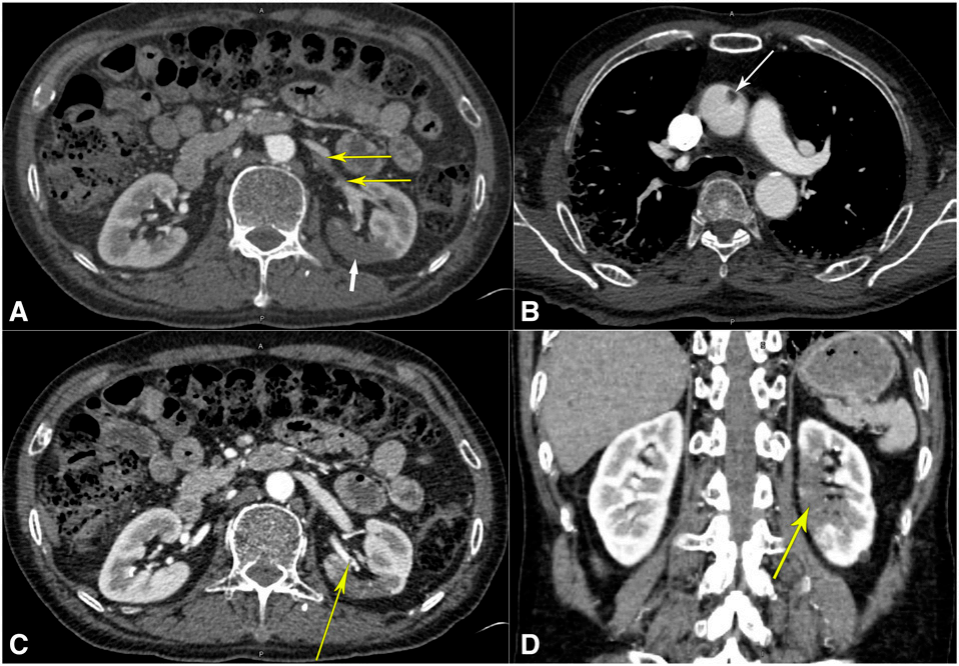
Computed tomogram with angiography. (**A**) Computed tomography angiogram of the abdomen showing left superior renal artery thrombi (thin yellow arrows) with infarcts in the posterior mid-pole of the left kidney (thick white arrow). (**B**) Computed tomography angiogram of the thorax showing the ascending aortic thrombus (arrow). (**C**) Computed tomography angiogram of the abdomen showing another view of the left superior renal artery thrombus (yellow arrow). (**D**) Computed tomography angiogram of the abdomen in the coronal view revealing the extent of the left renal infarction (yellow arrow). This figure appears in color at www.ajtmh.org.

However, he was readmitted 2 days later with worsened hypoxia. Vitals revealed he was afebrile at 98.9°F (37.1°C), blood pressure was 134/80 mmHg, tachycardic with a pulse of 112/minute, tachypneic at 34 breaths/minute, and hypoxic to 88% on 4 L/minute of oxygen via NC. Examination still noted bilateral crackles in all lung fields. Repeat investigations revealed positive COVID-19 test, persistent absolute lymphopenia, and further elevation of D-dimer, LDH, ferritin, and CRP (Table 1). A repeat chest X-ray showed worsened bilateral patchy opacities (Figure 1B). Saturations improved to 92% on 6 L/minute of oxygen supplementation. Hydroxychloroquine therapy was discontinued because of elevated QTc of 505 milliseconds. With worsening COVID-19 pneumonia, we initiated methylprednisolone 40 mg intravenously twice daily, along with lopinavir–ritonavir 400–100 mg orally twice daily and enoxaparin 40 mg subcutaneously twice daily. A computed tomography–pulmonary angiogram was negative for pulmonary embolism (PE) but revealed extensive multi-lobar ground-glass opacities (Figure 1C). Because of persistent dyspnea and hypoxia in the next 2 days, he was intermittently proned while on 6 L/minute oxygen via NC, and maintained saturation of 90–92%. Laboratory parameters were repeated intermittently as noted in Table 1. On day #9 since initial presentation, the patient complained of sharp left iliac fossa and left flank pain, which was high grade (8/10), constant, and radiating to his back, associated with nausea but no vomiting, diarrhea, dysuria, hematuria, fever, chills, or rigors. Urinalysis revealed clear, straw-colored, non-bloody urine with 30 mg/dL protein. An urgent computed tomography angiogram (CTA) revealed left superior renal artery thrombi with infarcts in the posterior mid-pole of the left kidney (Figure 2A, C, and D). The CTA also revealed ascending aortic thrombus (Figure 2B). Bilateral lower extremity ultrasound was negative for deep venous thrombosis (DVT). We initiated heparin intravenous infusion for therapeutic anticoagulation and discontinued enoxaparin. A loading dose of clopidogrel 300 mg was given and continued with 75 mg daily. On surgical review, no intervention was advised. The next day, the abdominal pain had resolved, but we discontinued lopinavir–ritonavir as the hepatic enzymes were rising. The oxygen requirement decreased to 4 L/minute by day #12 since presentation, and we switched to apixaban 5 mg orally twice daily for anticoagulation. The patient continued to steadily recover, with improving inflammatory and renal markers (Table 1). The urine output and electrolytes remained normal throughout (Table 1). By day #14 since initial presentation, the patient was with a saturation of 90% on room air at rest, only requiring supplemental oxygen on mobilization, and corticosteroids were discontinued. The next day, he was discharged home with home oxygen therapy and 3 months of apixaban and clopidogrel regimen.

## DISCUSSION

COVID-19 is caused by SARS-CoV-2, an enveloped beta coronavirus. Our knowledge regarding the pathophysiology of SARS-CoV-2 infection is still evolving. The SARS-CoV-2 infection activates an inflammatory response, releasing inflammatory mediators and activating the endothelium and hemostatic systems, with a concomitant increase of the von Willebrand factor and tissue factor.^2^ This manifests with a prominent elevation of D-dimer and fibrin/fibrinogen degradation products, whereas abnormalities in other coagulation parameters and platelet counts are relatively uncommon in the initial phases.^4,6,7^ Later, the complement pathway and cytokines like interleukin-6 (IL-6) are also involved, further activating the procoagulant pathway.^4^ In addition, SARS-CoV-2 has been detected in the pharynx, heart, liver, brain, kidney, and blood.^8^ It might infect endothelial cells via cluster of differentiation 13 (CD13) and angiotensin converting enzyme 2 (ACE2) receptors, resulting in diffuse endothelial inflammation and microvascular damage, which can result in widespread thrombosis; however, endothelial infection has been contested.^2,4,7,9–11^ Other proteins, such as transmembrane serine protease 2, sialic acid receptors, extracellular matrix metalloproteinase inducer (CD147 or basigin), and cathepsins B and L are all known as possible mediators facilitating SARS-CoV-2 entry in host cells, and are also expressed in endotheliocytes.^7^ This prothrombotic state may be further potentiated by an underlying viral hepatic dysfunction/damage with decreased antithrombin production.^2,6^ This results in disruption of the natural antithrombotic state.^4^ Occasionally, because of unclear mechanisms, the inflammatory response is severe, resulting in a dysregulated release of mediators like IL-6 (“cytokine storm”), resulting in magnified pathophysiology. Antiphospholipid antibodies (APLAs) have also been detected in some COVID-19 patients, which may lead to thrombotic events similar to antiphospholipid syndrome.^12^ Hence, we can infer how the interplay between the aforementioned factors leads to COVID-19–induced thromboembolic complications.

Renal infarction is relatively rare, with an incidence of 0.1–1.4%.^13,14^ Risk factors include male gender, hypertension, peripheral arterial disease, hyperlipidemia, and smoking.^14,15^ Etiology includes embolization, in situ thrombosis of the renal artery, renal artery dissection, fibromuscular dysplasia, hypercoagulability, and following endovascular interventions.^13–15^ Most patients have abdominal or flank pain, and, occasionally, fever, nausea, uncontrolled hypertension, hematuria, or acute renal failure. The rarity of the condition along with the nonspecific presentation makes diagnosis challenging.^13,15^ Imaging is essential for diagnosis, with contrast-enhanced computed tomogram (CT) being preferable; however, angiography remains gold standard.^13^

Before, in a series of two cases with renal infarcts in patients with COVID-19, one presented with acute kidney injury (AKI), whereas the other had AKI with associated bowel ischemia.^16^ It has also been found in a kidney transplant recipient without any arterial or venous thrombi.^17^ In another patient, the renal infarct was associated with renal artery thrombosis; however, the renal function remained normal, similar to our patient.^18^ Severe infections with viruses such as influenza, pandemic H5N1/H1N1 influenza, parvovirus B19, cytomegalovirus, Epstein–Barr virus, hepatitis C, dengue, and chikungunya have been associated with thromboembolism and associated renal dysfunction.^19–23^ COVID-19–associated aortic thrombosis has been found in association with PE, acute limb ischemia, DVT, acute mesenteric ischemia, and stroke.^24,25^ Severe and fatal cerebral venous thrombosis has also been noted.^26^

In our patient, we hypothesized that severe COVID-19 pneumonia induced a prothrombotic state, resulting in the ascending aortic thrombosis. This thrombus likely embolized, or there could have been an isolated neo-thrombosis in the renal artery as well. This thromboembolic pathology manifested with renal infarction. We treated our patient with hydroxychloroquine and later lopinavir–ritonavir as per existing institutional protocols; however, this regimen was later deemed controversial in the literature.^27–31^ Corticosteroid therapy can lower mortality.^32^ Prone positioning of awake, non-intubated patients may improve oxygenation.^33,34^ Antiphospholipid antibodies were not tested in our patient. For anticoagulation, the guidelines proposed by the anticoagulation forum, the American College of Cardiology, and the International Society of Thrombosis and Haemostasis were followed.^2,3,5^ For noncritically ill hospitalized patients, a standard regimen of venous thromboembolism prophylaxis is recommended.^2,3,5^ For critically ill patients, higher doses are recommended based on expert opinion.^3^ Regimens may include enoxaparin 0.5 mg/kg subcutaneous twice daily, heparin 7,500 units subcutaneous three times daily, or low-intensity heparin infusion.^3^ This was instituted in our patient’s second hospitalization. For the treatment of arterial thrombosis, the patient was put on a heparin infusion.^14,15^ On discharge, a 45- to 90-day course of therapeutic anticoagulation is recommended depending on risk.^2,3,5^

In conclusion, the thromboembolic consequences of COVID-19 can occasionally result in rare complications such as aortic thrombosis and renal infarction. During a pandemic, we advise that the physicians should maintain a high degree of clinical suspicion to diagnose rare manifestations of this novel disease for timely management.

## References

[1] Guan W 2020 Clinical characteristics of Coronavirus disease 2019 in China. N Engl J Med 382: 1708–1720. 32109013 10.1056/NEJMoa2002032 PMC7092819

[2] Bikdeli B Global COVID-19 Thrombosis Collaborative Group, Endorsed by the ISTH, NATF, ESVM, and the IUA, Supported by the ESC Working Group on Pulmonary Circulation and Right Ventricular Function, 2020 COVID-19 and thrombotic or thromboembolic disease: implications for prevention, antithrombotic therapy, and follow-up: JACC state-of-the-art review. J Am Coll Cardiol 75: 2950–2973. 32311448 10.1016/j.jacc.2020.04.031 PMC7164881

[3] Barnes GD 2020 Thromboembolism and anticoagulant therapy during the COVID-19 pandemic: interim clinical guidance from the anticoagulation forum. J Thromb Thrombolysis 50: 72–81. 32440883 10.1007/s11239-020-02138-z PMC7241581

[4] Connors JM Levy JH, 2020 COVID-19 and its implications for thrombosis and anticoagulation. Blood 135: 2033–2040. 32339221 10.1182/blood.2020006000 PMC7273827

[5] Thachil J Tang N Gando S Falanga A Cattaneo M Levi M Clark C Iba T, 2020 ISTH interim guidance on recognition and management of coagulopathy in COVID-19. J Thromb Haemost 18: 1023–1026. 32338827 10.1111/jth.14810 PMC9906133

[6] Han H Yang L Liu R Liu F Wu KL Li J Liu XH Zhu CL, 2020 Prominent changes in blood coagulation of patients with SARS-CoV-2 infection. Clin Chem Lab Med 58: 1116–1120. 32172226 10.1515/cclm-2020-0188

[7] Sardu C Gambardella J Morelli MB Wang X Marfella R Santulli G, 2020 Hypertension, thrombosis, kidney failure, and diabetes: is COVID-19 an endothelial disease? A comprehensive evaluation of clinical and basic evidence. J Clin Med 9: 1417. 10.3390/jcm9051417 PMC7290769 32403217

[8] Puelles VG 2020 Multiorgan and renal tropism of SARS-CoV-2. N Engl J Med 383: 590–592. 32402155 10.1056/NEJMc2011400 PMC7240771

[9] Varga Z Flammer AJ Steiger P Haberecker M Andermatt R Zinkernagel AS Mehra MR Schuepbach RA Ruschitzka F Moch H, 2020 Endothelial cell infection and endotheliitis in COVID-19. Lancet 395: 1417–1418. 32325026 10.1016/S0140-6736(20)30937-5 PMC7172722

[10] Goldsmith CS Miller SE Martines RB Bullock HA Zaki SR, 2020 Electron microscopy of SARS-CoV-2: a challenging task. Lancet 395: e99. 32442529 10.1016/S0140-6736(20)31188-0 PMC7237172

[11] Frelih M Erman A Wechtersbach K Pleško J Avšič-Županc T Kojc N, 2020 SARS-Cov-2 virions or ubiquitous cell structures? Actual dilemma in COVID-19 era. Kidney Int Rep 5: 1608–1610. 10.1016/j.ekir.2020.07.003. 32838080 PMC7362839

[12] Zhang Y 2020 Coagulopathy and antiphospholipid antibodies in patients with COVID-19. N Engl J Med 382: e38. 32268022 10.1056/NEJMc2007575 PMC7161262

[13] Caravaca-Fontán F Pampa Saico S Elías Triviño S Galeano Álvarez C Gomis Couto A Pecharromán de las Heras I Liaño F, 2016 Acute renal infarction: clinical characteristics and prognostic factors. Nefrologia 36: 141–148. 26698927 10.1016/j.nefro.2015.09.015

[14] Faucon AL Bobrie G Jannot AS Azarine A Plouin PF Azizi M Amar L, 2018 Cause of renal infarction: a retrospective analysis of 186 consecutive cases. J Hypertens 36: 634–640. 29045340 10.1097/HJH.0000000000001588

[15] Silverberg D Menes T Rimon U Salomon O Halak M, 2016 Acute renal artery occlusion: presentation, treatment, and outcome. J Vasc Surg 64: 1026–1032. 27345378 10.1016/j.jvs.2016.04.043

[16] Post A den Deurwaarder ESG Bakker SJL de Haas RJ van Meurs M Gansevoort RT Berger SP, 2020 Kidney infarction in patients with COVID-19. Am J Kidney Dis 76: 431–435. 32479921 10.1053/j.ajkd.2020.05.004 PMC7258815

[17] Xu JJ Samaha D Mondhe S Massicotte-Azarniouch D Knoll G Ruzicka M, 2020 Renal infarct in a COVID-19-positive kidney-pancreas transplant recipient. Am J Transplant 1–4. 10.1111/ajt.16089. PMC7300779 32483909

[18] Tascón GC 2020 Renal infarction in a patient with active COVID-19 infection. Nefrologia, 10.1016/j.nefro.2020.04.008.

[19] Goeijenbier M van Wissen M van de Weg C Jong E Gerdes VEA Meijers JCM Brandjes DPM van Gorp ECM, 2012 Review: viral infections and mechanisms of thrombosis and bleeding. J Med Virol 84: 1680–1696. 22930518 10.1002/jmv.23354 PMC7166625

[20] Canpolat N Topal N Civilibal M Caliskan S Sever L Kasapcopur O Baserer T Arisoy N, 2008 A case of catastrophic antiphospholipid syndrome in an adolescent girl with parvovirus B19 infection. Clin Pediatr (Phila) 47: 593–597. 18566354 10.1177/0009922808315216

[21] Baid S Pascual M Williams WW Tolkoff-Rubin N Johnson SM Collins B Chung RT Delmonico FL Cosimi AB Colvin RB, 1999 Renal thrombotic microangiopathy associated with anticardiolipin antibodies in hepatitis C-positive renal allograft recipients. J Am Soc Nephrol 10: 146–153. 9890320 10.1681/ASN.V101146

[22] Ramacciotti E 2019 Zika and chikungunya virus and risk for venous thromboembolism. Clin Appl Thromb Hemost 25: 1076029618821184. 30808213 10.1177/1076029618821184 PMC6714924

[23] Bunce PE High SM Nadjafi M Stanley K Liles WC Christian MD, 2011 Pandemic H1N1 influenza infection and vascular thrombosis. Clin Infect Dis 52: e14–e17. 21288835 10.1093/cid/ciq125

[24] Le Berre A Marteau V Emmerich J Zins M, 2020 Concomitant acute aortic thrombosis and pulmonary embolism complicating COVID-19 pneumonia. Diagn Interv Imaging 101: 321–322. 32334995 10.1016/j.diii.2020.04.003 PMC7161476

[25] Gomez-Arbelaez D Ibarra-Sanchez G Garcia-Gutierrez A Comanges-Yeboles A Ansuategui-Vicente M Gonzalez-Fajardo JA, 2020 COVID-19-Related aortic thrombosis: a report of four cases. Ann Vasc Surg 67: 10–13. 32474145 10.1016/j.avsg.2020.05.031 PMC7256515

[26] Cavalcanti DD 2020 Cerebral venous thrombosis associated with COVID-19. AJNR Am J Neuroradiol 41: 1370–1376. 32554424 10.3174/ajnr.A6644 PMC7658892

[27] Mukherjee A Ahmad M Frenia D, 2020 A Coronavirus disease 2019 (COVID-19) patient with multifocal pneumonia treated with hydroxychloroquine. Cureus 12: e7473. 32351851 10.7759/cureus.7473 PMC7187998

[28] Cao B 2020 A trial of lopinavir-ritonavir in adults hospitalized with severe COVID-19. N Engl J Med 382: 1787–1799. 32187464 10.1056/NEJMoa2001282 PMC7121492

[29] Geleris J 2020 Observational study of hydroxychloroquine in hospitalized patients with COVID-19. N Engl J Med 382: 2411–2418. 32379955 10.1056/NEJMoa2012410 PMC7224609

[30] Rosenberg ES 2020 Association of treatment with hydroxychloroquine or azithromycin with in-hospital mortality in patients with COVID-19 in New York state. JAMA 323: 2493–2502. 32392282 10.1001/jama.2020.8630 PMC7215635

[31] Arshad S Henry Ford COVID-19 Task Force, 2020 Treatment with hydroxychloroquine, azithromycin, and combination in patients hospitalized with COVID-19. Int J Infect Dis 97: 396–403. 32623082 10.1016/j.ijid.2020.06.099 PMC7330574

[32] Horby P RECOVERY Collaborative Group, 2020 Dexamethasone in hospitalized patients with COVID-19 - preliminary report. N Engl J Med, 10.1056/NEJMoa2021436. PMC7383595 32678530

[33] Thompson AE Ranard BL Wei Y Jelic S, 2020 Prone positioning in awake, nonintubated patients with COVID-19 hypoxemic respiratory failure. JAMA, 10.1001/jamainternmed.2020.3030. PMC7301298 32584946

[34] Elharrar X Trigui Y Dols AM Touchon F Martinez S Prud’homme E Papazian L, 2020 Use of prone positioning in nonintubated patients with COVID-19 and hypoxemic acute respiratory failure. JAMA 323: 2336–2338. 32412581 10.1001/jama.2020.8255 PMC7229532

